# Editorial: Evolution, pathogenesis, host interactions and therapeutic strategies against monkeypox virus

**DOI:** 10.3389/fcimb.2023.1303598

**Published:** 2023-10-12

**Authors:** Mehboob Hoque, Sneha Singh, Saadullah Khattak

**Affiliations:** ^1^ Applied Biochemistry Laboratory, Department of Biological Sciences, Aliah University, Kolkata, India; ^2^ Ophthalmology Vision and Anatomical Sciences Department, School of Medicine, Kresge Eye Institute, Wayne State University, Detroit, MI, United States; ^3^ Henan International Joint Laboratory of Nuclear Protein Regulation, School of Basic Medical Sciences, Henan University College of Medicine, Kaifeng, Henan, China

**Keywords:** monkeypox (MPX), orthopox virus, epidemiology, public health concerns, diagnosis, treatment

Considering the current global crisis of COVID-19, another viral pandemic might exacerbate concerns as well as the healthcare and socioeconomic repercussions. The present outbreak of monkeypox on a worldwide scale is unprecedented. Monkeypox is a zoonotic viral disease caused by a double-stranded DNA virus MPXV of the orthopox genus. This was first identified as a pox-like viral disease in monkeys in 1958, and the first case of human monkeypox was detected in the Democratic Republic of the Congo in 1970. Since then, occasional cases and minor outbreaks have been reported in non-endemic countries, with their re-emergence in 2022. On July 23, 2022, the World Health Organization (WHO) declared monkeypox a global public health emergency.

A perspective review by Shah et al. delineates the journey of monkeypox since the first human case, focusing on the reasons for the growing instances and methods to prevent the virus from spreading. The evolution of MPXV is governed by positive Darwinian selection that drives the adaptation of host interaction (Zhan et al.). The study using 156 coding genes encompassing more than 95% of the MPXV genome isolated from 1,500 samples found that the genes involved in host interaction and host determination (MPXVgp004, 010, 012, 014, 044, 098, 178, 188, and 191) evolved under positive Darwininan selection. Three recently reported missense substitution mutations (T/A426V in MPXVgp010, A423D in MPXVgp012, and S105L in MPXVgp191) were found to be critical for the virus’ adaptation to humans. Thus, monitoring the mutational landscape of the viral genes, particularly those undergoing positive selection for predicting viral transmission and virulence, is crucial.

As there are not many treatment options available for monkeypox disease, understanding the mechanisms of transmission and symptoms might help prevent the disease’s spread. Common disease symptoms include fever, swollen lymph nodes, muscular pains, headache, backache, and weariness. The appearance of rashes with blisters and crusts follows these symptoms. Although the symptoms typically develop 10 to 14 days after infection, they can appear up to 21 days. One to four days following the prodrome, there is a severe, deep-seated, vesicular, or pustular rash with central distribution. The face is most commonly afflicted, followed by the palms, soles, oral mucous membrane, and occasionally the genitalia and conjunctivae (Khattak et al.; Rampogu et al.). A meta-analysis by Rani et al. confirms that skin lesions contain the viral DNA that raises the risk of infection and potential transmission via direct skin-to-skin contact. The virus can spread to people by contact with the body fluids, skin lesions, and droplets of infected animals. Additionally, eating undercooked meat, being exposed to contaminated fomites, infected animals, eating bush meat, or wild game may all result in animal-to-human transmission. Human-to-human transmission can also occur when patients share the same household and consumables, or through healthcare contact (Rampogu et al.).

Identifying a disease at the right time is crucial for prevention and treatment. There are several methods available for the diagnosis of MPXV that include assays for IgM and IgG, Enzyme-Linked Immunosorbent Assay (ELISA), Polymerase Chain Reaction (PCR), electron microscopy, virus isolation, Immunofluorescence antibody test, and histopathology (Khattak et al.). Unfortunately, most of these techniques are equivocal and cannot distinguish MPXV disease from other poxvirus infections. However, an accurate diagnosis can be made via PCR analysis. A DNA oligonucleotide microarray that involves the TNF receptor gene crmB has been designed as an additional rapid method for identifying orthopoxviruses (Khattak et al.).

Fast-track therapeutic development is crucial for combating a disease with a high transmission rate. To this end, computational drug discovery and repurposing of existing drugs could be a viable tool for developing therapies against MPXV. In an attempt to identify a potential drug against the virus, Ajmal et al. performed *in silico* screening of 9000 FDA-approved compounds from the DrugBank database against MPXV protein thymidylate kinase. The researchers found three compounds that might bind and inhibit the viral target: DB16335, DB15796, and DB16250, with DB16335 being proposed as a possible medication that could help prevent MPXV. In separate research, the potential of curcumin derivatives as MPXV and smallpox virus inhibitors was investigated using molecular docking and dynamic simulations (Akash et al.). They identified twelve possible natural curcumin compounds with antiviral potential. Al Mashud et al. conducted a similar *in silico* investigation to evaluate the ability of O-rhamnosides and Kaempferol-o-rhamnosides derivatives to inhibit MPXV and Marburg virus. They discovered that the compounds L07, L08, and L09 exhibited better affinity for the target protein than the FDA-approved antiviral drug Cidofovir, suggesting their potential for antiviral treatment against MPXV and Marburg virus. However, before these molecules can be utilized as drugs, they must be thoroughly investigated in real-world situations and put through clinical trials.

Vaccines are the most potent protective measures against viral infections. The smallpox vaccines are effective against MPXV. The ACAM2000, a newer-generation smallpox vaccination, has received FDA approval for treating MPXV (Khattak et al.). However, the earlier generation of ACAM2000 may also be used off-label for the same purpose. Vaccinia immune globulin may be alternative post-exposure preventive measures if smallpox vaccination is unavailable. Translation of a novel drug or vaccine from laboratory to clinic requires time and should be tested in clinical trials. According to the study by Alorfi et al., only 10 interventional trials are registered at ClinicalTrials.gov. Of these, 4 trials were registered in Europe, 3 in America, and 3 in Africa. Forty percent of the registered trials were conducted for the JYNNEOS vaccine and 30% for Tecovirimat. The effectiveness and safety of the medications and vaccinations used to treat MPXV must thus be evaluated immediately through large-scale randomized clinical trials. Global concerted efforts must be made continuously to prepare for another epidemic.

## Author contributions

MH: Writing – original draft, Writing – review & editing. SS: Writing – review & editing. SK: Writing – original draft, Writing – review & editing.

